# The Emerging Role of CD8^+^ Tissue Resident Memory T (T_RM_) Cells in Antitumor Immunity: A Unique Functional Contribution of the CD103 Integrin

**DOI:** 10.3389/fimmu.2018.01904

**Published:** 2018-08-15

**Authors:** Stéphanie Corgnac, Marie Boutet, Maria Kfoury, Charles Naltet, Fathia Mami-Chouaib

**Affiliations:** INSERM UMR 1186, Integrative Tumor Immunology and Genetic Oncology, Gustave Roussy, EPHE, PSL, Fac. de Médecine – Univ. Paris-Sud, Université Paris-Saclay, Villejuif, France

**Keywords:** CD8 tissue resident memory T (T_RM_) cells, CD103 integrin, cytotoxic T lymphocytes, onco-immunology, cancer immunotherapy

## Abstract

Cancer immunotherapy is aimed at stimulating tumor-specific cytotoxic T lymphocytes and their subsequent trafficking so that they may reach, and persist in, the tumor microenvironment, recognizing and eliminating malignant target cells. Thus, characterization of the phenotype and effector functions of CD8^+^ T lymphocytes infiltrating human solid tumors is essential for better understanding and manipulating the local antitumor immune response, and for defining their contribution to the success of current cancer immunotherapy approaches. Accumulating evidence indicates that a substantial subpopulation of CD3^+^CD8^+^ tumor-infiltrating lymphocytes are tissue resident memory T (T_RM_) cells, and is emerging as an activated tumor-specific T-cell subset. These T_RM_ cells accumulate in various human cancer tissues, including non-small-cell lung carcinoma (NSCLC), ovarian and breast cancers, and are defined by expression of CD103 [α_E_(CD103)β_7_] and/or CD49a [α1(CD49a)β1] integrins, along with C-type lectin CD69, which most likely contribute to their residency characteristic. CD103 binds to the epithelial cell marker E-cadherin, thereby promoting retention of T_RM_ cells in epithelial tumor islets and maturation of cytotoxic immune synapse with specific cancer cells, resulting in T-cell receptor (TCR)-dependent target cell killing. Moreover, CD103 integrin triggers bidirectional signaling events that cooperate with TCR signals to enable T-cell migration and optimal cytokine production. Remarkably, T_RM_ cells infiltrating human NSCLC tumors also express inhibitory receptors such as programmed cell death-1, the neutralization of which, with blocking antibodies, enhances CD103-dependent TCR-mediated cytotoxicity toward autologous cancer cells. Thus, accumulation of T_RM_ cells at the tumor site explains the more favorable clinical outcome, and might be associated with the success of immune checkpoint blockade in a fraction of cancer patients.

## Introduction

CD8^+^ T lymphocytes play an essential role in defense against cancers through recognition by T-cell receptors (TCR) of specific antigenic peptides presented on the surface of malignant cells by major histocompatibility complex class I (MHC-I) molecules, and elimination of the tumor target, mainly by releasing the content of cytolytic granules containing perforin and granzymes. To destroy their target, cytotoxic T lymphocytes (CTL) must first migrate to the tumor site, infiltrate the tumor tissue, and interact with the cancer cell, to finally trigger effector functions leading to transformed cell eradication. Integrins and their ligands (1) play a crucial role in promoting antitumor T-cell activities by regulating T-cell migration and retention within the tumor, adhesion to antigen-presenting cells and co-stimulation resulting in CTL activation and functions (2). Cytokines and chemokines are also involved in coordinating circulation, homing, retention, and activation of T lymphocytes. Although some of them are known to contribute to tumor cell proliferation and dissemination by inhibiting tumor-specific T-cell responses, others promote infiltration and activation of T lymphocytes in a hostile tumor ecosystem, resulting in tumor cell destruction (3). In this regard, TGF-β, abundant in the tumor microenvironment, was reported to be an immunosuppressive factor used by malignant cells to escape from the immune response (4). This cytokine inhibits expression of lymphocyte-function-associated antigen-1 (LFA-1, also known as α_L_β_2_ or CD11a) integrin and LFA-1-mediated T-cell functions (5). Paradoxically, this cytokine induces CD103 (also known as α_E_β_7_ or HML-1) integrin expression on activated intraepithelial CD8^+^ T lymphocytes, and enhanced CD103-dependent T-cell adhesion and signaling (6, 7).

LFA-1 and CD103 are the predominant integrins expressed by intraepithelial T lymphocytes (IEL) and CD8^+^ tumor-infiltrating lymphocytes (TIL). While the contribution of LFA-1 and its ligand ICAM-1 (CD54) to TCR-mediated CTL activities is well documented (8), much less is known about the role of CD103 and its ligand, the epithelial cell marker E-cadherin, to T-cell-mediated cytolytic activity. CD103 has been associated with cytotoxicity of CD8^+^ T cells in several human pathologies, including graft-versus-host disease (GVHD) (9), allogeneic transplant rejection (10–12), autoimmune diseases (13, 14), and cancer (6, 15). This integrin, together with the activation marker CD69 and the integrin CD49a [also known as α_1_β_1_ or very late antigen-1 (VLA-1)], defines a recently identified subtype of CD8^+^ T lymphocytes called “tissue-resident memory T (T_RM_) cells,” possibly endowed with potent cytotoxic activities. Moreover, there is an emerging consensus that T_RM_ cells frequently accumulate in multiple human tumors, especially of epithelial origin, and play an essential role in tumor-specific T-cell responses and, likely, in control of malignant diseases. T_RM_ cells are also surrogate markers of the efficacy of cancer vaccines (16, 17), and a low number of this T-cell subset among TIL may correlate with failure of immune checkpoint blockade therapy in most cancer patients. In this review, we focus on CD8^+^ T_RM_ cells accumulating in human solid tumors, mainly non-small-cell lung carcinoma (NSCLC), and current insight implicating CD103 integrin in regulating T_RM_ functions and CTL-mediated antitumor immune responses, with potential prognosis and immunotherapeutic applications.

## Phenotypic and Molecular Features of T_RM_ Cells in Tumors

It is now generally agreed that a population of T_RM_ cells accumulates in tumors of epithelial origin, such as ovarian, pancreatic, colorectal, and lung tumors (15, 18–20), as well as those of non-epithelial origin, including malignant glioma and melanoma (21, 22). These T_RM_ cells express a broad range of integrins and chemokine receptors, probably involved in their migration to the tumor site, and may interfere with their egress from the tumor tissue. Transcriptional studies pointed to expression of CXCR3 and CXCR6 by T_RM_ cells infiltrating human lungs (23). Intratumoral T_RM_ cells express high levels of CCR5 and CCR6 chemokine receptors that may confer T-cell homing to the inflammatory tumor microenvironment (15). Moreover, CCR5 is recruited at the immune synapse formed between T cells and tumor target cells upon interaction of CD103 with E-cadherin, promoting retention of T_RM_ cells at the tumor site by inhibiting their sensitivity to a CCL5 chemotactic gradient (7). By contrast, T_RM_ cells do not express CX3CR1, a chemokine receptor that mediates transmigration through the endothelium, supporting the hypothesis that this T-cell population has reached its final destination and does not need to exit from the lung tissue (23). Lung tumor T_RM_ lack expression of lymph node homing receptors CCR7 and CD62L, as well as the receptor for sphingosine 1-phosphate, S1PR1 (15), which mediates the egress of T cells from lymphoid organs (24). Indeed, downregulation of SIPR1 appears to be a prerequisite for retention of CD8^+^CD103^+^ T_RM_ cells in peripheral tissues (25, 26).

With regard to adhesion/costimulatory molecules, the expression profile of intratumoral T_RM_ cells seems to be compatible with their capacity to reside in tumor tissue and their inability to recirculate in the bloodstream. In melanoma, CD8^+^ T_RM_ cells were found to co-express CD69, CD103, and VLA-1 (CD49a or α_1_β_1_ integrin), with the latter reported to cause long-term retention of activated T cells in peripheral tissues (27). Human lung tumor CD8^+^ T_RM_ cells are characterized by downregulation of CD28 and upregulation of CD69 and CD103 and CD49a integrins, which are most likely induced by TGF-β in the tumor microenvironment (15, 28). TGF-β plays a pivotal role in formation and maintenance of T_RM_, at least in part *via* induction of CD103. Indeed, TGF-β is directly involved in CD103 expression in tumor-specific T cells upon engagement of TCR with specific tumor peptide–MHC-I complexes (7), through binding of Smad2/3 and NFAT-1 transcription factors to promoter and enhancer elements of the *ITGAE* gene, which encodes the CD103 (α_E_) subunit (29). This cytokine is also involved in dampening expression of the LFA-1 integrin on TIL, thus participating in T-cell residency within the tumor (15, 30). In LCMV chronic infection, but not acute infection, TGF-β signaling inhibits migration of CD8^+^ effector T lymphocytes from the spleen to the gut by dampening expression of integrin α_4_β_7_ during the formation phase of T_RM_ cells (31). Consequently, CD8^+^ Tgfbr2^−/−^ T cells migrate normally to the intestine, but their retention in the gut epithelium is impaired. In contrast, TGF-β signaling does not impact α_4_β_7_ integrin expression and T-cell migration to the gut after acute bacterial infection (32). Moreover, E-cadherin, which is downregulated by TGF-β in cancer cells during epithelial-to-mesenchymal transition [for a review see Ref. (33)], appeared to promote accumulation of a subset of CD8^+^ memory T cells in murine submandibular glands by a mechanism independent of CD103 (34). This cytokine has been identified as a potential therapeutic target in cancer because of its role in supporting tumor progression and in inducing immunosuppression. In this regard, it has been shown that targeting the TGF-β pathway inhibits tumor growth by promoting antitumor immunity associated with increased CD8^+^ T-cell numbers (35). However, the consequence of such cancer immunotherapy approaches on T_RM_ cells, the maintenance of which is dependent of TGF-β, has not been addressed.

T-cell inhibitory receptors are important for maintaining self-tolerance and regulating the immune response in peripheral tissues (36). Among these immune checkpoints, cytotoxic T-lymphocyte-associated antigen (CTLA)-4 and Tim-3 appeared to be associated with tumor antigen-specific CD8^+^ T-cell dysfunction in melanoma patients (37). CD103^+^ T_RM_ cells have been shown to express a wide range of inhibitory receptors, such as CTLA-4, Tim-3, and programmed cell death-1 (PD-1), associated with their capacity to maintain peripheral tolerance (25, 38). Data from our group and other groups revealed that intratumoral CD8^+^CD103^+^ T_RM_ cells frequently express PD-1, Tim-3, and Lag-3, which are likely involved in their exhausted state and their dysfunctioning at the tumor site (15, 28, 39, 40). Notably, TGF-β is also involved in PD-1 induction on CD8^+^ T cells, contributing to T-cell anergy and a sustained tolerance (41). Neutralization of TGF-β results in downregulation of PD-1 expression in T cells causing graft rejection. Mechanistically, PD-1 is regulated by the NFATc1 transcription factor (42), and is enhanced by a TGF-β/SMAD3-dependent signaling pathway (43). Expression of PD-1 on TIL is described as a biomarker of CD8^+^ tumor-reactive T cells in cancer patients (44). Thus, the PD-1^+^ status of tumor T_RM_ cells suggests that they are enriched with antigen-specific CD8^+^ T cells that may be used as targets in cancer immunotherapy.

Alongside upregulation of genes encoding PD-1, CTLA-4 and Tim-3, CD8^+^ TIL display increased expression levels of genes encoding transcription factors EGR1 and Nr4a2 (25, 38), as well BATF and NAB1, suggesting a role in T_RM_ establishment in the tumor (28). CD8^+^CD103^+^ TIL also express an increased level of T-bet (45) and the Runx3 transcription factor, which programs their residency in tumors (46). Indeed, *Runx3* deficiency impaired TIL accumulation without affecting migration to the tumor, associated with an increase in tumor growth. By contrast, *KLF2* transcription factor was diminished in T_RM_ cells from human TIL^hi^ tumors (28), while Notch activity appeared to be required for maintenance of CD103^+^ T_RM_ cells in mouse tumors (23). Therefore, additional studies are needed to better characterize the transcriptional features of CD8^+^CD103^+^ T_RM_ cells in human tumors, and transcriptional factors that govern their residency in malignant tissues. Overall, the T_RM_ cell subset is characterized by a Runx3^+^, Notch^+^, Hobit^+^, Blimp1^+^, BATF^+^, EOMES^neg^, and Tbet^low^ transcription factor profile (23, 46–49) and is defined by the surface expression of CD103, CD49a, and CD69 [for reviews see Ref. (50–52)]. It also expresses the inhibitory receptors PD-1, CTLA-4, and Tim-3 (15, 38, 53), and is promoted by particular route of immunization targeting tissue dendritic cells (17, 54, 55) and specific environmental factors mainly TGF-β, IL-33, and IL-15 (56–59).

## Functional Activities of Intratumoral T_RM_ Cells

Thus far, little is known about CD8^+^CD103^+^ T_RM_ functions in tumor tissues. Immune checkpoint expression by CD103^+^ TIL suggested that CD8^+^ T_RM_ cells in tumors are enriched with tumor antigen-specific CTL. These T cells were found to express transcripts encoding products linked to cytotoxic functions of CD8^+^ T lymphocytes, including *IFNG, GZMA, GZMB, SEMA7A, KLRB1, CCL3, STAT1, RAB27A, IL21R*, and *FKBP1A* (28). Expression of granzyme A, granzyme B, and perforin by CD8^+^CD103^+^ TIL was also observed at the protein level, together with the CD107a (LAMP-1) degranulation marker and the Ki-67 proliferation marker (15, 28, 45, 60).

Functional studies showed that CD8^+^CD103^+^ TIL are able to secrete inflammatory cytokines, including interferon (IFN)γ and TNFα (28, 46). Moreover, interaction of CD103 with E-cadherin on tumor target cells optimizes cytokine release, since siRNA targeting E-cadherin partially inhibited IFNγ production (61). Cytotoxicity experiments indicated that freshly isolated CD103^+^ TIL were able to kill autologous tumor cells following neutralization of the PD-1–PD-L1 interaction with anti-PD-1 or anti-PD-L1 blocking antibodies (15). This cytotoxic activity is most likely mediated by CD103^+^ T cells, since anti-CD103 neutralizing monoclonal antibodies (mAb) compromise this function. Consistently, cytotoxicity of CD103^+^ T-cell clones toward autologous E-cadherin^+^ tumor cells is inhibited anti-CD103 blocking mAb (6). Another noteworthy aspect of our contribution to the field is the demonstration that CD103 is an important molecule required for polarization of cytotoxic granules at the immune synapse formed between CTL clones and autologous tumor cells, and that siRNA targeting E-cadherin inhibited TCR-mediated target cell killing (6). Moreover, CD103 contributes to recruitment of CD103^+^ T_RM_ cells within epithelial tumor islets, and intratumoral early T-cell signaling (30).

A role for the VLA-1 integrin in the differentiation and functions of T_RM_ cells was reported in a mouse tumor model (27). VLA-1^+^ T cells, co-expressing or not CD103, secreted high levels of IFNγ upon re-stimulation, and this cytokine production was impaired by anti-VLA-1 or anti-CD103 mAb. Moreover, blockade of VLA-1 or CD103 severely compromised control of tumor growth *in vivo*. Similar studies revealed that CD8^+^CD103^+^ T_RM_ cells accumulate and protect mice against melanoma in a CD103-dependent manner, and these T_RM_ cells play a pivotal role in perpetuating antitumor immunity (22). Conversely, it has been reported that anti-latency-associated peptide (LAP) antibodies targeting the LAP/TGF-β complex induce a decrease in CD8^+^CD103^+^ T cells in mouse spleen and lymph nodes, and that this peculiar T-cell subset displays a tolerogenic feature (62). Murine CD8^+^CD103^+^ regulatory T cells have also been described in autoimmune diseases where they are induced by TGF-β and display suppressive activities independently of granzyme B (63). Moreover, CD8^+^CD103^+^ T cells are crucial for prevention of chronic GVHD lupus in mice by suppressing T helper and B cell responses through a non-cytotoxic mechanism involving TGF-β and IL-10 signals (64). However, further studies are needed to permit the distinction between human CD8^+^CD103^+^ CTL and CD8^+^CD103^+^ T regulatory cells, even though granzyme B expression appears as a good marker, and determine the exact contribution of both subsets in autoimmune [for a review see Ref. (65)] and cancer diseases.

## Bidirectional Signaling of CD103 Dictates its Activation and Functions

Integrins are heterodimeric transmembrane receptors that mediate cell-extracellular matrix adhesion and cell–cell interactions (2). Among a family of 24 members (1), the CD103 integrin, formed by α_E_ (CD103) and β_7_ subunits, is exclusively expressed by leukocytes, in particular IEL (66), psoriatic skin epidermal CD8^+^ T cells (67), cervico-vaginal antigen-specific CTL (68), and CD8^+^ T lymphocytes infiltrating various human tumors (6, 18–20, 60, 69). The restricted distribution of the CD103 integrin is attributed to expression of the α_E_ subunit, since the β_7_ subunit is widely expressed in T cells (70).

On naive T lymphocytes, integrins have weak affinity for their ligands. However, stimulation of T lymphocytes through TCR or chemokine receptors initiates an “inside-out” signal that induces integrin activation by triggering integrin-extended conformation and clustering, thereby enhancing their affinity for their ligands. Firm adhesion of integrins to their ligands triggers an “outside-in” signal that has costimulatory functions in TCR signaling, thereby contributing to T-cell activation, migration, and cytotoxicity (71–73). Until recently, the signaling pathways of CD103 integrin and the molecules involved in its bidirectional activation were not clearly elucidated. Like the other integrins, CD103 activation is regulated by TCR engagement. In this context, it has been shown that cross-linking of TCR on IEL or cell treatment with phorbol myristate acetate increased the avidity of CD103 for E-cadherin and provided a mechanism for lymphocyte adherence and activation (74). Furthermore, the CCR9 ligand, CCL25, induced CD103-mediated adhesion of CD8^+^ IEL to E-cadherin, suggesting a role for this chemokine receptor/chemokine pair in promoting functions of CD103 *via* inside-out signaling (75). Similarly, the CCL7 chemokine has been shown to favor adhesion and retention of CD103-expressing T cells during renal allograft rejection, by promoting the adhesive properties of CD103 (76).

**Figure 1:**
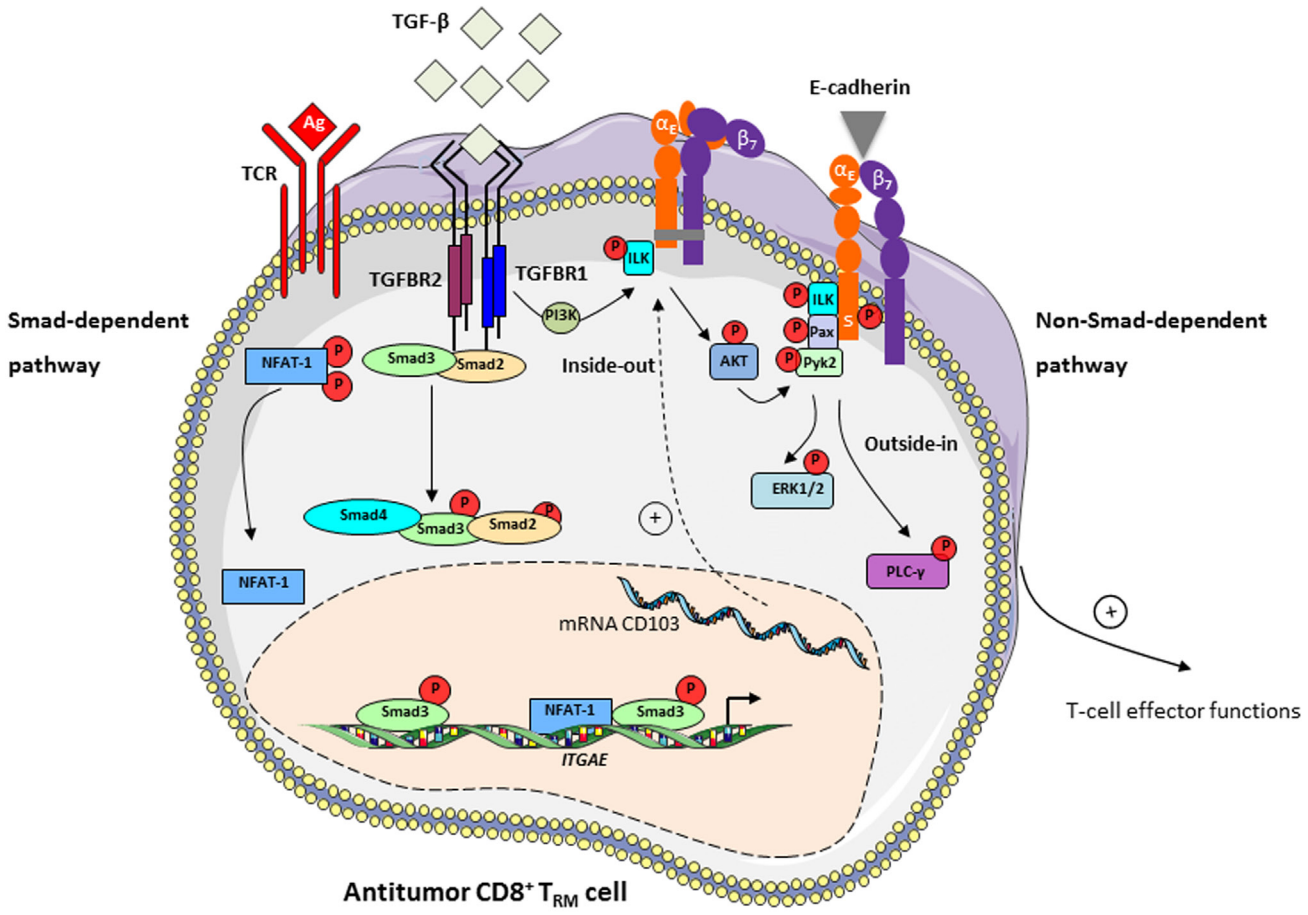
TGF-β induces CD103 expression in tumor-specific T cells and participates in integrin bidirectional signaling. *Left*: TGF-β controls CD103 expression in tumor antigen (Ag)-specific T cells upon interaction of T-cell receptor (TCR) with specific tumor peptide–major histocompatibility complex class I complexes, *via* a Smad-dependent pathway. TGF-β binds to TGFBR at the surface of CD8^+^ T lymphocytes and leads to recruitment and phosphorylation of Smad2 and Smad3 and their subsequent nuclear translocation. Transcription factors NFAT-1, translocated into the nucleus upon TCR engagement, and Smad2/3 bind to promoter and enhancer elements of the *ITGAE* gene, which encodes the CD103 (α_E_) subunit, and activates CD103 expression (29). *Right*: TGF-β participates in CD103 intracellular signaling *via* a non-Smad-dependent pathway. Interaction of TGF-β with TGFBR on CD8^+^CD103^+^ T_RM_ cells induces recruitment and phosphorylation of integrin-linked kinase (ILK). Phosphorylated (P)-ILK interacts with the CD103 subunit intracellular domain, resulting in phosphorylation of protein kinase B/AKT and initiating integrin inside-out signaling leading to activation of CD103 (30). CD103-E-cadherin tight adhesion initiates an outside-in signal by promoting phosphorylation of paxillin (Pax) and Pyk2, and subsequent binding of phosphorylated-paxillin to the α_E_ subunit tail where a phosphorylatable Ser (S) in the ES^1163^IRKAQL motif plays an important role (80). Adhesive interaction of E-cadherin with CD103 also triggers activation of PI3K/extracellular signal-regulated kinase (ERK) and phospholipase C/PKC pathways (60), providing intracellular signals that promote CD8^+^ T_RM_ effector functions, including actin cytoskeleton reorganization, T-cell spreading and migration, cytokine release and polarized exocytosis of cytotoxic granules leading to target cell destruction.

TGF-β is responsible for inducing CD103 integrin in CD8^+^ T lymphocytes (6, 77) by regulating expression of both *ITGAE* (29, 78) and *ITGB7* (79) genes encoding α_E_ and β_7_ chains, respectively. In addition, in contrast to all other integrins, TGF-β regulates CD103 activation and signaling within epithelial tissues (Figure 1). Indeed, we previously demonstrated that the interaction of TGF-β with its receptors TGFBR on the surface of CD8^+^CD103^+^ T cells induces recruitment and phosphorylation of integrin-linked kinase (ILK) by TGFBR1 (activin receptor-like kinase-5) (30). We further showed that phosphorylated-ILK interacted with the CD103 subunit intracellular domain, resulting in phosphorylation of protein kinase B (PKB)/AKT, thereby initiating integrin inside-out signaling leading to activation of CD103 and strengthening of CD103-E-cadherin adhesion.

The mechanism regulating the CD103 outside-in signaling pathway is not fully understood. Studies from our group have shown that CD103-E-cadherin tight adhesion initiates an outside-in signal by promoting phosphorylation of the focal-adhesion-associated adaptor protein paxillin and proline-rich tyrosine kinase-2 (Pyk2), and subsequent binding of phosphorylated-paxillin to the CD103 subunit tail (80). In addition, the adhesive interaction of E-cadherin with CD103 on TIL triggers phosphorylation of extracellular signal-regulated kinases 1 and 2 (ERK1/2) and phospholipase C γ1 proteins, providing intracellular signals that promote CTL effector functions (60). These studies emphasize a unique costimulatory role of the CD103 integrin in activation of tumor-specific CTL, by triggering polarization of cytotoxic granules at the immune synapse and subsequent TCR-mediated cytotoxicity (60), and in proliferation of CD103^+^ thymocyte cells (81). Engagement of CD103 with E-cadherin also determines cell shape and motility of CD103^+^ lymphocytes (82), and recruitment of CD8^+^ T_RM_ cells within epithelial tumor islets, in an actin-polymerization-dependent fashion (30, 80). Moreover, TGF-β enhances T-cell adhesion and movement toward tumor regions by increasing CD103 expression levels and promoting intracellular T-cell signals leading to integrin activation (30). CD103 also contributes to retention of T_RM_ cell subpopulations by interacting with E-cadherin and mediating arrest of T lymphocytes on epithelial tissues (32, 61). Thus, CD103 appears to be a unique integrin for adjusting T-cell adhesion and migratory potential in a TGF-β-rich tumor microenvironment, as well as retention of tumor-specific CD8^+^ T_RM_ cells and local antitumor effector functions (Figure 1).

## Prognostic Value of T_RM_ Cells in Human Cancers

CD8^+^CD103^+^ T_RM_ cells have emerged as predictive markers of patient survival in several malignant diseases, including ovarian, lung, endometrial, and breast cancers (15, 20, 28, 83, 84). Indeed, in a large cohort of high-grade serous ovarian cancers (20) and a cohort of early-stage NSCLC (15), an enhanced CD103^+^ TIL subset correlated with improved patient survival. CD103^+^ TIL were also associated with a favorable prognosis in urothelial cell carcinoma of the bladder, and could represent a favorable prognostic predictor of overall and recurrence-free survival (83). In that retrospective study, CD8^+^ T cells were identified as the principal cellular sources of CD103, and the density of intratumoral CD103^+^ cells was inversely associated with tumor size. More recent studies also defined the CD103 integrin as a biomarker of good prognosis in cohorts of breast (85) and lung cancer (17, 28, 84). Notably, T_RM_ infiltration in lung cancer correlated with better clinical outcome in both univariate and multivariate analyses, independently of CD8^+^ T cells (17). In addition, high numbers of intratumoral CD103^+^ TIL were significantly associated with prolonged disease-free survival and overall survival in patients with pulmonary squamous cell carcinoma, but not in those with pulmonary adenocarcinoma (84). The epithelial localization of CD103^+^ TIL has an even more significant predictive value compared to the stromal location, suggesting that intraepithelial CD8^+^CD103^+^ cells encompass a higher proportion of tumor-specific T_RM_ cells (15, 85). This intratumoral positioning of CD103^+^ TIL was correlated with expression of E-cadherin on tumor cells in bladder cancer (83), but not in ovarian or breast cancer (20, 85). Moreover, this predominant location in intratumoral regions, rather than in the stroma, was associated with the capacity of CD103 to promote recruitment of TIL in epithelial tumor islets (30). Thus, T_RM_ cells appear to be key components in antitumor immunity, and their presence at the tumor site predicts a favorable prognosis in many cancers of different histological types. Paradoxically, their dominant expression of checkpoint receptors suggests that may be functionally exhausted. However, their location in close contact with tumor cells, their ability to proliferate *in situ*, to produce granzyme B and other cytotoxic molecules and pro-inflammatory cytokines, support the hypothesis that T_RM_ cells are enriched in tumor-specific CD8^+^ T cells that could trigger specific cytotoxic activity toward target cells in physiological conditions and following neutralization of PD-1–PD-L1 interaction, as we demonstrated *ex vivo* (15) and possibly also during anti-PD-1/anti-PD-L1 cancer immunotherapy. Accordantly, recent studies revealed an expansion of CD8^+^CD103^+^ T_RM_ cells during anti-PD-1 treatment in melanoma (86).

## Concluding Remarks

**Figure 2:**
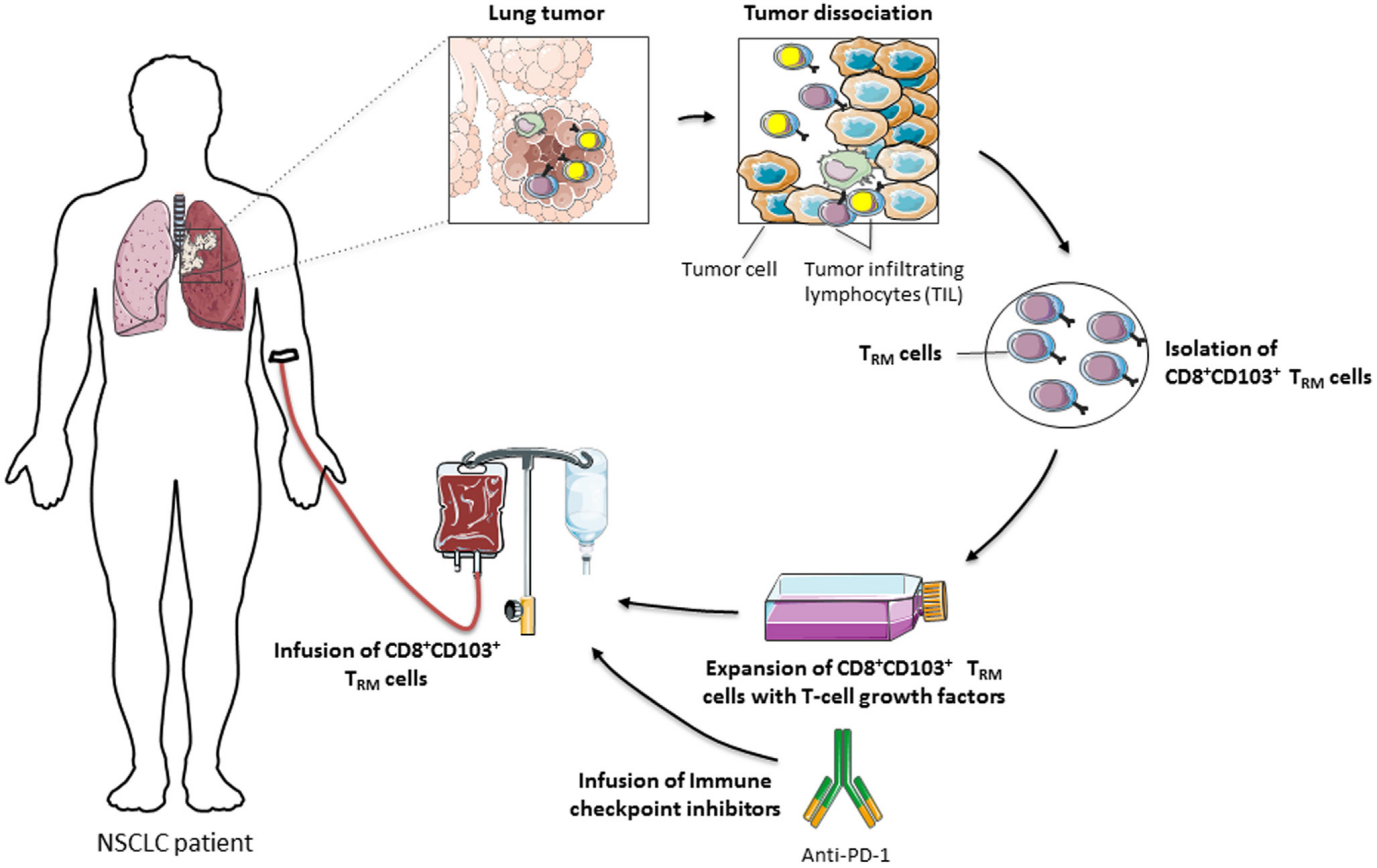
Adoptive transfer of CD8^+^CD103^+^ T_RM_ cells for cancer immunotherapy. CD8^+^CD103^+^ T_RM_ cells, suspected to express tumor-reactive T-cell receptors, are isolated from tumor-infiltrating lymphocytes (TIL), amplified *ex vivo* in the presence of T-cell growth factors, including IL-2, and then reinjected into cancer patients, in combination or not with immune checkpoint inhibitors, such as anti-programmed cell death-1, to reverse T-cell exhaustion and optimize the antitumor cytotoxic T lymphocyte response.

Overall, CD8^+^ T_RM_ cells that accumulate in human tumor lesions appear to be important effectors in antitumor CTL responses. Their retention within the tumor ecosystem may control tumor growth and explain more favorable prognoses in certain cancer patients. Moreover, CD103 emerges as a key molecule in CD8^+^ T_RM_ activation, the expression of which is probably adjusted in the tumor microenvironment by TGF-β. This integrin not only promotes T-cell adhesion to target cells through interaction with its unique known ligand E-cadherin but also provides positive signals triggering diverse T-cell effector functions, such as spreading, migration, proliferation, and cytotoxicity (Figure 1). Nevertheless, additional studies and tools are required to further decipher CD103 structure and bidirectional signaling, and to determine whether this integrin also undergoes conformational changes within the tumor ecosystem in order to control the affinity to its ligand E-cadherin and to regulate its functional properties. In this regard, identification of new partners and associated molecules controlling integrin intracellular signals and regulating the dynamics of CD103 are essential in order to optimize the antitumor reactivity of CD8^+^ T_RM_ cells. They would also help to determine the true contribution of CD8^+^CD103^+^ T_RM_ cells and the identified costimulatory molecules in the success of immune checkpoint blockade immunotherapies in a minor subpopulation of cancer patients, and to improve current T-cell-based cancer immunotherapeutic approaches such as adoptive T-cell therapies (Figure 2).

## Author Contributions

SC, MB, and FM-C coordinated the writing of the manuscript. SC, MB, MK, CN, and FM-C participated in drafting and editing the text and figures. All authors gave final approval to the version submitted.

## Conflict of Interest Statement

The authors declare that the research was conducted in the absence of any commercial or financial relationships that could be construed as a potential conflict of interest.

## Abbreviations

CTL, cytotoxic T lymphocytes; CTLA, cytotoxic T-lymphocyte-associated antigen; PD-1, programmed cell death-1; IFN, interferon; LFA-1, lymphocyte-function-associated antigen-1; mAb, monoclonal antibodies; NSCLC, non-small-cell lung carcinoma; MHC-I, major histocompatibility complex class I; TCR, T-cell receptor; TIL, tumor-infiltrating lymphocytes.
